# Self-oriented affective empathy is associated with increased negative affect

**DOI:** 10.1038/s41598-025-09860-9

**Published:** 2025-07-30

**Authors:** Mareike J. Hülsemann, Aleksandra Kaurin, Bianca Kollmann, Andrea Chmitorz, Kira F. Ahrens, Charlotte Schenk, Michael M. Plichta, Beat Lutz, Ulrike Basten, Christian J. Fiebach, Raffael Kalisch, Klaus Lieb, Andreas Reif, Oliver Tüscher, Henning Hermes, Daniel Schunk, Michèle Wessa

**Affiliations:** 1 Department of Clinical Psychology and Neuropsychology, Institute for Psychology, Johannes Gutenberg-University Mainz, Mainz, Germany; 2 Clinical Child and Adolescent Psychology and Psychotherapy, Institute of Psychology, University of Wuppertal, Wuppertal, Germany; 3 Leibniz Institute for Resilience Research (gGmbH), Wallstraße 7, 55122 Mainz, Germany; 4 Department of Social Work, Education and Nursing Sciences, Esslingen University, Esslingen, Germany; 5 Department of Psychiatry, Psychosomatic Medicine and Psychotherapy, University Hospital, Goethe University Frankfurt, Frankfurt, Germany; 6 Institute of Physiological Chemistry, University Medical Center Mainz, Mainz, Germany; 7 Department of Psychology, RPTU Kaiserslautern-Landau, Landau, Germany; 8 Department of Psychology, Goethe University Frankfurt, Frankfurt, Germany; 9 Brain Imaging Center, Goethe University, Frankfurt, Germany; 10 Department of Psychiatry and Psychotherapy, University Medical Center Mainz, Mainz, Germany; 11 Ifo Institute Munich, Munich, Germany; 12 Department of Economics, Johannes Gutenberg-University Mainz, Mainz, Germany; 13 Division of Cancer Survivorship and Psychological Resilience, German Cancer Research Center (DKFZ) Heidelberg, Heidelberg, Germany; 14 Department of Neuropsychologgy and Psychological Resilience Research, Central Institute of Mental Health, Mannheim, Germany; 15 DKFZ Hector Cancer Institute at the University Medical Center Mannheim, Mannheim, Germany; 16 Department of Psychiatry, Psychotherapy and Psychosomatic Medicine, Martin-Luther University Halle-Wittenberg, Halle, Germany

**Keywords:** Cognitive empathy, Affective empathy, Anxiety, Depression, Latent profile analysis, Replication, Human behaviour, Quality of life, Risk factors, Lifestyle modification

## Abstract

An increasing body of research suggests that empathic traits at high levels may predict negative affectivity. Here, we investigate the combinatory and differential role of affective (personal distress, empathic concern) and cognitive (perspective taking) facets of empathy for their contribution to negative affectivity in two general population samples (N_1_ = 259, N_2_ = 938). A latent profile analysis revealed four combinatory groups of affective and cognitive empathic facets (i.e., high affective high cognitive [A+/C+], high affective low cognitive [A+/C−], low affective high cognitive [A−/C+], low affective low cognitive [A−/C−]). These groups were differentially associated with negative affectivity, showing that greater affective empathy was associated with increased negative affect. Moreover, moderation and subsidiary simple slopes analyses demonstrated that self-oriented affective empathy (personal distress) was generally positively associated with depression and anxiety. In case of depressive symptomatology, this correlation was lower under circumstances of high cognitive empathy, but only in the larger, second sample. Other-oriented affective empathy (empathic concern) was not related to negative affect. Our findings suggest that enhanced self-focused affective empathy may be associated with exaggerated involvement in the emotional experience of others, with the potential to reduce the negative correlation of accurate emotion recognition with negative affect.

## Introduction

Empathy, the ability to feel into others emotional state^1^, has traditionally been seen as a generally positive characteristic that enables us to build relationships and to navigate in social settings. Previous research showed that high levels of empathy are associated with positive interpersonal outcomes. For example, empathy promotes communication skills that lead to better understanding between health care providers and patients, leading to more effective therapeutic change^2,3^. Furthermore, empathy has been primarily positively associated with high-quality relationships and prosocial behavior^4–6^. However, findings are mixed and an emerging body of research puts forth, that empathy may not be a uniformly positive attribute^7^. Two recent meta-analyses accumulate evidence, that empathy is positively related to symptoms of depression^8^ and anxiety^9^. Importantly, both meta-analyses showed that affective empathy was associated with negative affect, whereas cognitive empathy was not. Despite the lack of a uniform definition of empathy^10^, theories often differentiate between affective empathy and cognitive empathy^11,12^. The former comprises emotional contagion, while the latter describes a form of understanding other’s emotional states^13^. This dichotomy emphasizes that empathy helps us to both affectively share and cognitively recognize emotions of others^14,15^. Yet sharing other’s aversive feelings may cause discomfort and anxiety^16^.

A prominent example for this regard is emotional exhaustion in caregivers. Andreychik^17^ has shown that negative empathy, conceptualized as the process of contagion with negative emotions, was related to greater burnout levels and secondary traumatic stress in experienced and regular providers for direct client care. Similar results were obtained in board-certified physicians^18^. A recent study demonstrated that being frontline worker in a collective crisis (COVID-19 pandemic) was not only associated with perceived stress and burnout symptoms, but also general empathy levels^19^. Research on the collapse of compassion has shown that the presence of a large number of people in distress can lead to a decrease in empathetic responses^20^. One reason for this phenomenon may be the negative emotions that arise with empathy^21^. Furthermore, it has been shown that the number of personal crises positively correlates with self-reported general empathy^22^. Thus, the omnipresence of personal and collective crises could place those with high affective empathy in a vulnerable position. Particularly in times when there are many situations of collective compassion, it is important to be able to regulate one’s empathic reactions, as these have been found to be predictive of mental health problems in an adolescent sample^23^.

Smith^11^ has formalized the idea that enhanced empathic traits may be predictive of psychopathological conditions within a typology of *empathy disorders*. Four empathy types emerge from combinations of high versus low levels of affective and cognitive empathy (high affective high cognitive [A+/C+], high affective low cognitive [A+/C−], low affective high cognitive [A−/C+], low affective low cognitive [A−/C−]). Different lines of research suggest that cognitive and affective empathy are distinct dimensions. For example, lesion studies have identified distinct anatomical structures for both systems of empathic traits^12^. Furthermore, research could not substantiate a predictive relationship between affective empathy and the ability to correctly identify another’s affective state (e.g.^24^). Assuming that cognitive and affective empathy are distinct dimensions they may also be differentially linked to negative affectivity. A cluster analysis in a sample of medical students revealed three different subgroups, one with low affective (emotional concern and personal distress) and high cognitive empathy (perspective taking), a second with high affective and high cognitive empathy, and a third with high empathetic concern and perspective taking but low personal distress^25^. These clusters were differentially related to the Big Five personality traits, with those high in personal distress having the highest levels of neuroticism, i.e., emotional lability^26^. This study did, however, not relate clusters to mental disturbances, such as symptoms of depression or anxiety and results are limited to a very particular population (medical students).

The aim of the present study was to address two research questions using an adult sample from the general population. First, are there distinguishable subgroups in the general population that differ in their levels of affective and cognitive empathy? That is, can we find the postulated distinction and independency of affective and cognitive facets of empathy in self-reported measures of empathy. To answer this question, we ran latent profile analyses (LPA) in order to reveal the hypothesized combinatory groups of affective empathy (i.e. personal distress and empathic concern) and cognitive empathy (i.e. perspective taking) proposed by Smith^11^. We validated the predictive value and thus the relevance of the identified profiles by comparing them with respect to levels of depression and anxiety. Second, how do the two facets of empathy, independently of and in combination with each other, relate to negative affect? That is, can we find evidence for the notion, that affective, but not cognitive empathy is a risk factor for mental health difficulties? Furthermore, is high cognitive empathy a protective factor in that it buffers the potentially negative effect of affective empathy on mental health? To answer these questions, we examined whether high cognitive empathy was accompanied by a reduced likelihood to experience negative affect under circumstances of extreme affective empathy. We used two independent population-based samples, that both provided self-report measures of affective and cognitive empathy, depression, and anxiety to cross validate the respective results.

## Results

### Study 1

#### Sample characteristics

The first sample was assessed in the framework of an educational intervention study in primary schools^27^. The data analyzed in this paper were not part of the regular data collection of this intervention study but a separate follow-up survey for the study presented here. The sample consisted of N = 259 parents (232 (89.6%) females and 27 (10.4%) males). Mean age was 42.3 (SD = 6.2) years, ranging from 26 to 75 years. All the participants were parents (primarily mothers) of children in primary school.

Men showed significantly lower scores for empathic concern compared to women (2.43 ± 0.76 vs. 2.86 ± 0.54; *F*(1,29.15) = 8.56, *p* = .007, ω_p_^2^ = 0.050). No significant gender differences were observed for personal distress (*F*(1,257) = 0.77, *p* = .381, ω_p_^2^ = − 0.001) or perspective taking (*F*(1,257) = 2.09, *p* = .149, ω_p_^2^ = 0.004). Moreover, no significant gender differences were found for any of our dependent variables (depression: *F*(1,257) = 0.46, *p* = .497, ω_p_^2^ = − 0.002; anxiety: *F*(1,256) = 0.82, *p* = .367, ω_p_^2^ = − 0.001). See Table 1 for means and standard deviations. Pearson correlation coefficients indicated that age was not correlated with any facet of empathy nor with any of the outcome variables (r_personal distress_ = 0.05, *p* = .406; r_empathic concern_ = − 0.08, *p* = .228; r_perspective taking_ < 0.01, *p* = .986; r_depression_ = − 0.06, *p* = .350; r_anxiety_ = − 0.09, *p* = .157).

**Table 1:** Descriptive statistics of and correlations between all variables included in the moderation analyses.

Variable	Study 1					Study 2				
	M	SD	1	2	3	M	SD	1	2	3
Depression	0.51	0.39	− 0.19	0.37	0.13	0.39	0.34	− 0.14	0.28	0.06
Anxiety	0.83	0.45	− 0.22	0.55	0.17	0.56	0.51	− 0.09	0.32	0.10
Predictor										
(1) Perspective taking	2.42	0.63	–	− 0.12	0.32	2.48	0.66	–	− 0.16	0.32
(2) Personal distress	1.69	0.62		–	0.33	1.72	0.63		–	0.37
(3) Empathic concern	2.82	0.58			–	2.56	0.68			–

Values for depression and anxiety are not directly comparable as different instruments were used in the two studies. All instruments used a scale from 0 to 3. Instruments for depression (CES-D in Study 1 and PHQ-9 in Study 2) assess current symptoms within the last weeks. In Study 1 dispositional anxiety (STAI-T) is assessed while in Study 2 current symptoms of anxiety are assessed (GHQ-28 subscale). N_1_ = 259 for Study 1 and N_2_ = 938 for Study 2.

#### Latent profiles of individuals based on cognitive and affective empathy traits

**Fig. 1:**
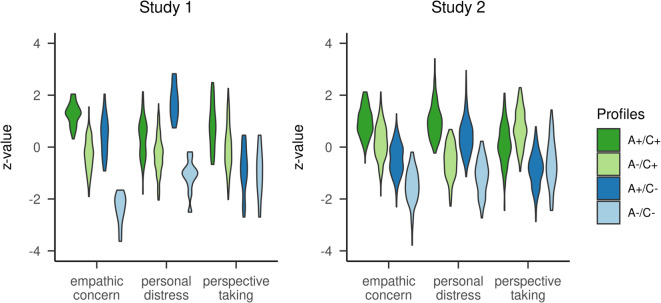
Profiles identified by the LPA. Violin plots of estimated mean standardized values of the profiles based on affective components of empathy (empathic concern and personal distress) and cognitive empathy (perspective taking) within each of the four identified profiles. N_1_ = 259 for Study 1 and N_2_ = 938 for Study 2.

A four-profile solution of the LPA models was chosen because it had the lowest Bayesian information criterion (BIC) in absolute terms (Table 2). According to the BIC, there was positive evidence that the four-profile solution had a better fit than the three-profile solution. Importantly, only the four-profile solution—but not the two- or three-profile solution—had a meaningfully decreased BIC in comparison to the solution with no separate profiles^28^. The four-profile solution was also supported by the bootstrapped likelihood ratio test (BLRT) and the entropy measure^29^. Furthermore, the four profiles correspond to those predicted by Smith, thereby providing this solution with theoretical justification. We identified all four profiles postulated by Smith^11^, cf. Fig. 1. The largest profile (66.0%, n = 171) consisted of people scoring low on affective components of empathy and relatively high on the cognitive component of empathy (A−/C+). The smallest profile (4.2%, n = 11) was made up of individuals scoring low on all facets of empathy (A−/C−). Another rather small profile (10.8%, n = 28) was detected with higher values on the affective component of empathy, yet lower values on the cognitive component of empathy (A+/C−). In addition, we found a profile of people (18.9%, n = 49), scoring high on both facets of empathy (A+/C+). The profiles differed significantly in terms of empathic concern (*F*(3,37.28) = 200.33, *p* < .001, ω_p_^2^ = 0.698), personal distress (*F*(3,255) = 76.51, *p* < .001, ω_p_^2^ = 0.467), and perspective taking (*F*(3,255) = 28.53, *p* < .001, ω_p_^2^ = 0.242). Post hoc t-tests showed that each profile was significantly different from the others (all p_bonf_ < .001), except A+/C− and A−/C−, which did not differ in terms of perspective taking (p_bonf_ > .999). The naming of the profiles was mainly based on personal distress and perspective taking, with less emphasis on empathic concern (see “Discussion” section). Age differences between the four profiles were significant, but not substantial (*F*(3,28.65) = 3.30, *p* = .034, ω_p_^2^ = 0.034). Subjects with higher scores on cognitive empathy, irrespective of their expression of the affective facets of empathy, were on average younger than subjects with lower scores on cognitive empathy (median [range]: A±/C+: 42 [26–58] years vs. A±/C−: 45 [32–75] years). Since men and women differed with respect to empathic concern, we compared the proportion of males and females for each profile relative to the total amount of each subset of participants. Females were more likely in profile A+/C+ than expected, but less likely in profile A−/C− than expected. Contrary to that, males were more likely in profile A−/C− than expected, but less likely in profile A+/C+ than expected. Gender differences in the distribution across latent profiles were significant (χ^2^(3, n = 259) = 25.25, *p* < .001).

**Table 2:** Fit of the latent profile models by increasing numbers of profiles for personal distress, emotional concern, and empathic accuracy.

Profiles	Study 1						Study 2					
	Log-likelihood	AIC	BIC	BLRT(p)	Entropy	n per class	Log-likelihood	AIC	BIC	BLRT(p)	Entropy	n per class
1	− 1101.01	2214.02	2235.37	–	1.00	259	− 3991.39	7994.78	8023.85	–	1.00	938
2	− 1087.62	2195.25	2230.82	0.01	0.39	134/125	− 3907.66	7835.32	7883.75	0.01	0.60	648/290
3	− 1074.21	2176.41	2226.21	0.01	0.53	35/116/108	− 3879.95	7787.90	7855.71	0.01	0.49	402/271/265
4	− 1061.23	2158.47	2222.49	0.01	0.69	28/11/49/171	− 3856.06	7748.12	7835.30	0.01	0.52	232/348/233/125
5	− 1056.71	2157.41	2235.67	0.19	0.60	19/13/108/56/63	− 3839.28	7722.56	7829.12	0.01	0.59	297/246/19/100/276
6	− 1053.86	2159.73	2252.20	0.40	0.60	21/61/93/11/38/35	− 3835.14	7722.27	7848.21	0.10	0.55	138/99/57/93/396/155

N_1_ = 259 for Study 1 and N_2_ = 938 for Study 2.

*AIC* Akaike information criterion, *BIC* Bayesian Information criterion, *BLRT* Bootstrapped likelihood ratio test.

#### External validation of the latent profiles

**Fig. 2:**
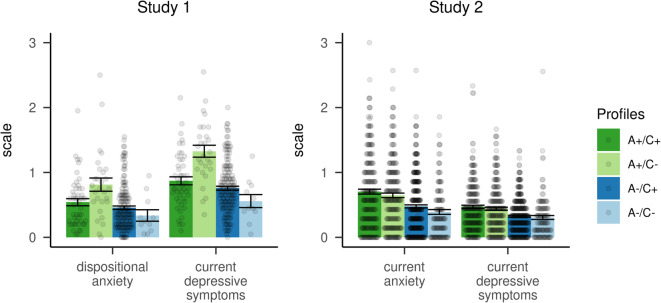
Latent profiles and their association with anxiety and depression. Bar plots represent the mean ± SE and violin plots represent single subject data (N_1_ = 259, N_2_ = 938). High affective empathy (A+/C±) is associated with higher reported symptoms of anxiety and depression. In Study 1 this effect was eliminated when subjects also had high levels of cognitive empathy. In Study 2, cognitive empathy did not act as protective factor. Note an important difference between both studies: In Study 1 dispositional anxiety (STAI-T) is assessed while Study 2 assesses current symptoms of anxiety (GHQ-28) within the last weeks. Both studies assess current symptoms of depression within the last weeks (Study 1: CES-D, Study 2: PHQ-9).

We found a significant main effect of latent profile for both dependent variables assessed (depression: *F*(3,255) = 7.99, *p* < .001, ω_p_^2^ = 0.075; anxiety: *F*(3,254) = 17.26, *p* < .001, ω_p_^2^ = 0.159; Fig. 2). The (A+/C−) profile with high affective and low cognitive levels of empathy had significantly higher symptoms of depression and anxiety than all other profiles (depression: A+/C− vs. A+/C+: *p*_bonf_ = .016; A+/C− vs. A−/C+: *p*_bonf_ < .001; A+/C− vs. A−/C−: *p*_bonf_ = .003; anxiety: all *p*_bonf_ < .001). These other three profiles did not significantly differ from each other regarding symptoms of negative affect (depression: A−/C− vs. A+/C+: *p*_bonf_ = .636; A−/C− vs. A−/C+: *p*_bonf_ > .999; A+/C+ vs. A−/C+: *p*_bonf_ = .998; anxiety: A−/C− vs. A+/C+: *p*_bonf_ = .140; A−/C− vs. A−/C+: *p*_bonf_ = .720; A+/C+ vs. A−/C+: *p*_bonf_ = .550).

#### Relationship of empathy and negative affectivity

The LPA analysis showed that high affective empathy was associated with negative affect if cognitive empathy was relatively low (profile A+/C−), but not if cognitive empathy was relatively high (profile A+/C+). Therefore, we analyzed whether perspective taking, personal distress, and empathic concern were linearly related to depression and anxiety, independent of the previously detected latent profiles. In a first step we analyzed the main effects of both empathy facets. In a second step, we tested whether the interaction of both had additional explanatory value, i.e., whether one facet moderates the effect of the other. See Table 1 for descriptive statistics of and correlations between all included variables. Detailed results of these analyses are shown in Table 3 for depression and Table 4 for anxiety.

**Table 3:** Summary of the results of the moderation analyses with the criterion depression.

	Study 1					Study 2				
	R^2^	*β* [95% CI]	SE	*t*	*p*	R^2^	*β* [95% CI]	SE	*t*	*p*
Dependent variable: depression										
Step 1	0.16					0.09				
Perspective taking (PT)		− 0.18 [− 0.30, − 0.05]	0.06	− 2.82	.005		− 0.09 [− 0.16, − 0.02]	0.03	− 2.57	.010
Personal distress (PD)		0.32 [0.20, 0.44]	0.06	5.13	< .001		0.28 [0.21, 0.35]	0.04	7.85	< .001
Empathic concern (EC)		0.08 [− 0.05, 0.21]	0.07	1.24	.215		− 0.02 [− 0.09, 0.05]	0.04	− 0.47	.638
Step 2	0.16					0.10				
Perspective taking (PT)		− 0.17 [− 0.30, − 0.05]	0.06	− 2.76	.006		− 0.09 [− 0.16, − 0.02]	0.03	− 2.68	.007
Personal distress (PD)		0.32 [0.20, 0.44]	0.06	5.09	< .001		0.27 [0.20, 0.34]	0.04	7.66	< .001
Empathic concern (EC)		0.08 [− 0.05, 0.21]	0.07	1.24	.217		− 0.02 [− 0.09, 0.05]	0.04	− 0.47	.642
Interaction (PT × PD)		− 0.03 [− 0.14, 0.09]	0.06	− 0.46	.648		− 0.09 [− 0.14, − 0.02]	0.03	− 2.63	.009
Interaction (PT × EC)		0.03 [− 0.08, 0.13]	0.05	0.48	.634		0.02 [− 0.04, 0.08]	0.03	0.70	.482
Conditional effects of personal distress at values of perspective taking										
− 1 SD_PT_		–	–	–	–		0.35 [0.26, 0.44]	0.05	7.79	< .001
+ 1 SD_PT_		–	–	–	–		0.19 [0.10, 0.29]	0.05	3.96	< .001

For significant interactions the conditional effects of affective empathy (personal distress) at − 1 SD- and + 1 SD-values of the moderator cognitive empathy (perspective taking) are shown. Both studies, examined current symptoms of depression.

N_1_ = 259, N_2_ = 938.

**Table 4:** Summary of the moderation analyses with the criterion anxiety.

	Study 1					Study 2				
	R^2^	*β* [95% CI]	SE	*t*	*p*	R^2^	*β* [95% CI]	SE	*t*	*p*
Dependent variable: anxiety										
Step 1	0.33					0.10				
Perspective taking (PT)		− 0.18 [− 0.29, − 0.07]	0.06	− 3.25	.001		− 0.04 [− 0.11, 0.03]	0.03	− 1.22	.223
Personal distress (PD)		0.51 [0.40, 0.62]	0.06	9.04	< .001		0.31 [0.24, 0.38]	0.04	8.89	< .001
Empathic concern (EC)		0.06 [− 0.05, 0.18]	0.06	1.05	.296		0.00 [− 0.07, 0.07]	0.04	0.09	.925
Step 2	0.33					0.11				
Perspective taking (PT)		− 0.18 [− 0.29, − 0.07]	0.06	− 3.18	.002		− 0.05 [− 0.11, 0.02]	0.03	− 1.35	.177
Personal distress (PD)		0.51 [0.40, 0.62]	0.06	8.98	< .001		0.31 [0.24, 0.37]	0.04	8.71	< .001
Empathic concern (EC)		0.06 [− 0.05, 0.18]	0.06	1.04	.297		0.00 [− 0.07, 0.07]	0.04	0.04	.971
Interaction (PT × PD)		− 0.03 [− 0.13, 0.07]	0.05	− 0.56	.575		− 0.04 [− 0.10, 0.02]	0.03	− 1.18	.238
Interaction (PT × EC)		0.03 [− 0.07, 0.12]	0.05	0.55	.584		− 0.03 [− 0.09, 0.03]	0.03	− 0.96	.338

Conditional effects of affective empathy at values of the moderator cognitive empathy are not shown, because none of the interactions were significant. Study 1 assessed dispositional anxiety, while Study 2 measured current symptoms of anxiety.

N_1_ = 259, N_2_ = 938.

*Depression.* In a first step, we examined how perspective taking, personal distress, and empathic concern were independently associated with depressive symptoms. The overall model was significant (*F*(3,255) = 16.40, *p* < .001) and explained 16.17% of the variance in depressive symptoms. Perspective taking (β = − 0.18, *p* = .005) and personal distress (β = 0.32, *p* < .001) did significantly correlate with depressive symptoms, while empathic concern did not (β = 0.08, *p* = .215). In the second step, we investigated whether cognitive and affective empathy modulated each other, by including the two-way interaction terms. This did not significantly improve the goodness-of-fit of the model (*F*(2,253) = 0.16, *p* = .851).

*Anxiety.* In relation to dispositional anxiety, we found perspective taking (β = − 0.18, *p* = .001) and personal distress (β = 0.51, *p* < .001) to be significant. Empathic concern did not significantly correlate with dispositional anxiety (β = 0.06, *p* = .296). The overall model was significant (*F*(3,254) = 41.52, *p* < .001) and explained 32.90% of the variance in dispositional anxiety. Including the interaction terms of cognitive and affective empathy in the second step did not significantly improve the goodness-of-fit of the model (*F*(2,252) = 0.23, *p* = .796).

### Study 2

#### Sample characteristics

The second sample came from the Longitudinal Resilience Assessment (LORA) study^30^. This is a convenience sample of initially healthy individuals drawn from the general population of the cities of Frankfurt and Mainz, Germany. We included the baseline assessment from LORA, resulting in a final sample of N = 938 adults (591 (63.0%) females and 345 (36.8%) males). Mean age was 28.6 years (SD = 7.8), ranging from 18 to 50 years.

In this sample, men showed significantly lower scores for both affective facets of empathy compared to women (empathic concern: 2.29 ± 0.66 vs. 2.72 ± 0.64, *F*(1,934) = 95.56, *p* < .001, ω_p_^2^ = 0.092; personal distress: 1.49 ± 0.59 vs. 1.85 ± 0.61, *F*(1,934) = 78.72, *p* < .001, ω_p_^2^ = 0.077). Again, no significant gender differences were observed for perspective taking (*F*(1,934) = 0.60, *p* = .440, ω_p_^2^ = 0.000). Moreover, no relevant gender difference were found for depression (*F*(1,934) = 0.55, *p* = .460, ω_p_^2^ = 0.000) or anxiety (*F*(1,815.54) = 4.22, *p* = .040, ω_p_^2^ = 0.003). See Table 1 for means and standard deviations.

Pearson correlation coefficients indicated that age was very weakly correlated with personal distress (r_personal distress_ = − 0.08, *p* = .014) and depression (r_depression_ = − 0.08, *p* = .013). Neither empathic concern or perspective taking nor the outcome variable anxiety were significantly associated with age (r_empathic concern_ = − 0.04, *p* = .179; r_perspective taking_ > 0.99, *p* = .285; r_anxiety_ = − 0.06, *p* = .055). The detected sizes of all correlations resemble those found in the first sample.

#### Latent profiles of individuals based on cognitive and affective empathy traits

We replicated the four-profile solution found in Study 1. This solution was the best as it had the meaningfully lowest BIC (Table 2). The number and characteristics of the empathy facets correspond to those postulated by Smith^11^, thereby offering a theoretical justification. The five-profile solution did not have a meaningfully decreased BIC compared to the four-profile solution^28^. Although the BLRT would also justify a five-profile solution, and the entropy for the five-profile solution was higher than for the four-profile solution, this would have resulted in a very small fifth profile (2.0%, n = 19) without a clear theoretical interpretation^29^. With the four-profile solution we identified the same profiles as in Study 1 (Fig. 1). Again, the largest profile (37.1%, n = 348) was made up of individuals with lower values on the affective component of empathy, yet higher values on the cognitive component of empathy (A−/C+). As in Study 1, the smallest profile (13.3%, n = 125) consisted of people scoring low on all scales (A−/C−). In addition, we found a profile of people (24.7%, n = 232), scoring high on both facets of empathy (A+/C+) and a profile (24.8%, n = 233) comprised of people with higher values on the affective dimension of empathy and lower values in the cognitive dimension (A+/C−). The profiles differed significantly in terms of empathic concern (*F*(3,934) = 482.62, *p* < .001, ω_p_^2^ = 0.606), personal distress (*F*(3,934) = 404.34, *p* < .001, ω_p_^2^ = 0.563), and perspective taking (*F*(3,399.62) = 289.81, *p* < .001, ω_p_^2^ = 0.480). Post hoc t-tests showed that each profile was significantly different from the others (all p_bonf_ ≤ .024). As in Study 1, the naming of the profiles was mainly based on personal distress and perspective taking, with less emphasis on empathic concern (see “Discussion” section). There were no substantial age differences between the four profiles (*F*(3,932) = 2.81, *p* = .038, ω_p_^2^ = 0.006). We compared the proportion of males and females for each profile relative to the total amount of each subset of participants. As in Study 1, females were more likely in profile A+/C+ than expected, but less likely in profile A−/C− than expected. Again, for males the opposite pattern was true. Gender differences in the distribution across latent profiles were significant (χ^2^(3, n = 938) = 84.94, *p* < .001).

#### External validation of the latent profiles

We found a significant main effect of latent profile for depression (*F*(3,392.51) = 12.61, *p* < .001, ω_p_^2^ = 0.037) and for anxiety (*F*(3,416.91) = 17.31, *p* < .001, ω_p_^2^ = 0.049) (Fig. 2). Both profiles with high affective empathy traits, irrespective of the extent of cognitive empathy, showed the strongest associations with negative affect and generally differed from both other profiles (depression: all *p*_bonf_ < .002; anxiety: all *p*_bonf_ < .001). (A+/C+) and (A+/C−) did however, not differ from each other (depression: *p*_bonf_ > .999; anxiety: *p*_bonf_ > .999). Similarly, there was no significant difference between profiles (A−/C+) and (A−/C−) with regards to negative affect (depression: *p*_bonf_ > .999; anxiety: *p*_bonf_ > .576).

#### Relationship of empathy and negative affectivity

For the regression model of negative affectivity, we exactly replicated the analyses conducted in the first sample. See Table 1 for descriptive statistics of and correlations between all included variables. Detailed results of these analyses are shown in Table 3 for depression and Table 4 for anxiety.

*Depression.* In a first step, we examined how perspective taking, personal distress, and empathic concern were uniquely associated with depressive symptoms. The overall model was significant (*F*(3,934) = 30.86, *p* < .001) and explained 9.02% of the variance in depressive symptoms. In the second step, we investigated whether cognitive and affective empathy modulated each other, by including the two-way interaction terms between cognitive and affective empathy. This significantly improved the goodness-of-fit of the model (*F*(2,932) = 3.45, *p* = .032), which then explained 9.69% of the variance in depression symptoms. Here, in contrast to Study 1, the interaction of perspective taking and personal distress was significantly related to depressive symptoms (β = − 0.09, *p* = .009). Simple slope analyses revealed that high cognitive empathy was accompanied by a decreased association between affective empathy and negative affect, whereas it was increased for low cognitive empathy. The interaction of perspective taking and empathic concern was not significant (β = 0.02, *p* = .482). Replicating the findings of Study 1, perspective taking (β = − 0.09, *p* = .007, step 2) and personal distress (β = 0.27, *p* < .001, step 2) additionally did significantly predict depressive symptoms independent of each other. Again, empathic concern did not significantly predict depressive symptoms (β = − 0.02, *p* = .642).

*Anxiety.* In Study 2 cognitive empathy itself did not significantly predict current symptoms of anxiety (β = − 0.04, *p* = .223). However, as in study 1, personal distress (β = 0.31, *p* < .001) did and empathic concern (β = 0.00, *p* = .925) did not significantly predict anxiety. The overall model was significant (*F*(3,934) = 35.94, *p* < .001) and explained 10.35% of the variance in anxiety. Including the interaction terms between cognitive and affective empathy in the second step did not significantly improve the goodness-of-fit of the model (*F*(2,932) = 1.66, *p* = .191). In contrast to Study 1, in this study we did not assess dispositional anxiety but rather symptoms of anxiety over the past few weeks.

## Discussion

Although empathy is a trait that has been discussed with regards to positive individual and interpersonal outcomes, recent findings raised doubts about the uniformity of beneficial effects of empathy. The present study investigated under which circumstances empathic traits are associated with increased individual susceptibility to experience negative affect in two large general population samples. We identified four empathy types characterized by all possible combinations of high and low affective and cognitive empathy. These were differentially associated with experiences of negative affectivity. We found that particularly high levels of negative arousal in response to the unpleasant experiences of others (i.e., personal distress) had detrimental consequences for one’s well-being, whereas other-oriented feelings of compassion (i.e., empathic concern) were not associated with anxiety or depression. The cognitive ability to understand the emotions of others had a small but negative correlation with negative affect. Our analyses did not reveal robust interaction effects between affective and cognitive empathy. However, we found evidence that the positive correlation between affective empathy and depressive symptoms may be attenuated when cognitive empathy skills are high.

The first aim of the present analyses was to determine whether there are distinguishable empathy types in the general population that differ in their affective and cognitive levels of empathy. We found the four subgroups postulated by Smith^11^ in both samples, underpinning the notion, that these two constructs are independent dimensions of empathy^11,12^. The dichotomy of affective and cognitive empathy also finds support in neuroimaging studies, revealing differentiable activation patterns for both systems^31^. In interpreting the latent profiles, we mainly relied on personal distress and perspective taking as indicators of affective and cognitive empathy, respectively. Supporting the independence of affective and cognitive empathy, personal distress and perspective taking were only weakly correlated. In contrast, these two facets of empathy correlated positively with empathic concern. Comparable correlations are reported by Kim and Han^32^ and Stosic et al.^33^. Furthermore, empathic concern was reliably highest in the (A+/C+) profile and lowest in the (A−/C−) profile, while profiles with different levels of personal distress and perspective taking (A+/C− and A−/C+) showed intermediate levels of empathic concern. This may indicate that empathic concern is not a pure indicator of affective empathy but contains both cognitive and affective components. Although empathic concern and personal distress are both classified as facets of affective empathy, empathic concern is an other-oriented construct, whereas personal distress is a self-oriented construct^34^. Previous studies have shown that personal distress and empathic concern are associated with activations in different brain regions during social situations and are differentially predictive of behavior^35,36^. Isrealashvili et al.^37^ found that empathic concern, but not personal distress, was positively associated with emotion recognition in others, underlining the involvement of cognitive processes in empathic concern. The role of empathic reactivity in the cognitive recognition of others’ emotions has been debated. Early conceptualizations proposed that the recognition of others’ emotions typically leads to either states of vicarious sharing of emotions via compassionate feelings of concern for another person or less convenient states of personal distress^38–40^. A newer line of evidence suggests that the shared experience of others emotional states is a crucial prerequisite to accurately recognize and label another’s state^16,41^. In contrast to the latter perspective, our results, particularly of the (A−/C+) profile, suggest that an accurate affect recognition can occur without personally experiencing these emotional states. However, as our results rely on cross-sectional and self-report data such a conclusion is highly preliminary. The proposed causal relationships need to be experimentally pitted against each other to further inform this debate.

We found that the (A+/C−) profile was strongly associated with negative affectivity, having the highest scores for depression and anxiety. In Study 1 this group differed from all other groups, which themselves did not show distinguishable depressive or anxious symptoms. That is, affective empathy was linked to negative affect, but only when it was not accompanied by high levels of cognitive empathy. In Study 2 we replicated this finding, but here, the (A+/C+) profile showed a similarly elevated negative affect. That is, in the more representative and larger replication sample, cognitive empathy did not mitigate the positive correlation between affective empathy and internalizing symptoms. As in Study 1, both profiles with low affective empathy showed comparably low negative affect. Our finding of personal distress being negatively related to psychological well-being, perspective taking to be positively related to well-being and empathic concern not being related to well-being, replicates findings of Diongi et al.^42^, who studied a sample of health care providers. Our results furthermore conceptually replicate the findings of Kim and Han^32^, who evaluated how personal distress is associated with other personality traits in three large samples. They found that, in contrast to empathic concern, personal distress was associated amongst others with depression, ruminative coping, neuroticism, and self-criticism.

Using moderation analyses, we tested whether cognitive empathy can attenuate the correlation between affective empathy and negative affect. Self-oriented affective empathy (personal distress) was strongly and reliably associated with mental health difficulties, indicated by elevated symptoms of anxiety and depression, while other-oriented affective empathy (empathic concern) was not. This is in line with previous studies on adolescents, reporting that personal distress is positively associated with emotional symptoms, but empathic concern is not^43^. Although cognitive empathy showed a weak but consistent association with lower levels of depression in both samples of the present paper, it elicits mixed findings regarding anxiety, not only in our samples but also in the literature. A recent meta-analysis did not find clear evidence for empathic accuracy, a facet of cognitive empathy, to be associated with clinical conditions of depression or anxiety^44^. Furthermore, a negative correlation between cognitive empathy and psychopathology could not be found in adolescents^45^. Importantly, we found no general moderating effect of cognitive empathy that mitigated the positive relationship of affective empathy and negative affect. However, in the larger sample of Study 2, this moderation was present regarding depressive symptoms. Overall, the results of Study 1 were replicated in Study 2, while the explanatory value of the models showed a regression to the mean, with overall lower effect sizes in the larger second sample compared to the first sample.

Anxiety in Study 2 and depression in both samples were assessed as state measures, representing mood and symptom clusters within the previous weeks. In contrast to that, anxiety in Study 1 was assessed as trait measure. Such an approach differs from the assessment of stable interindividual differences, in a way that it does not provide information about chronicity levels of a condition and associated changes in feelings, thoughts and actions. As previous studies suggested, the level of symptom chronicity, however, may be an important boundary condition to empathic behaviors. From research in professions that demand high levels of empathy it is known that over time individuals may develop signs of lessened empathy^46^. With this in mind, it is possible that under conditions of extreme concern for others (i.e., high levels of empathic concern), individuals are likely to move into a state of compassion fatigue, in which initial feelings of warmth may gradually give way to the experience of personal distress^7,46^. Future studies should therefore control for the duration of symptoms. This will help to determine whether the differential use of affective empathic strategies is related to the severity of depression and anxiety.

Furthermore, in accordance with previous studies^47^, personal distress showed a substantial overlap with symptoms of dispositional anxiety, mirrored by a strong main effect of personal distress in the relation to anxiety. It may reflect methodological difficulties in disentangling the exclusive contribution of related constructs in a regression model. Furthermore, it may be difficult for individuals to distinguish between both affective responses, as feelings of anxiety may be very proximal to experiences of personal distress because of vicariously shared emotions.

An important limitation is the cross-sectional nature of our data, which limits causal inference. Without longitudinal or experimental data, we cannot determine whether empathy actually influences negative affect. The causal relationship may be reversed, or both constructs may be correlated due to confounding factors. Another limitation of this study lies in the use of self-reports. Self-reported and behavioral measures of empathy are known to correlate to only small extents^48^. One possibility is that such a divergence mirrors a methodological artifact in the sense that differences in introspective abilities and levels of social desirability lower the validity of self-reports. However, Melchers et al.^48^ conclude, that self-report measures are valid and exchangeable, while behavioral measures seem to assess specific empathy aspects. A third limitation refers to the different composition of the two samples analyzed in the present study. The results of Study 1 should be interpreted with caution, as the sample size for latent profile analysis is rather small (N = 259). A minimum sample size of 500 is recommended^29^. Whereas the first sample included 90% females, more precisely mothers, the second sample had a more balanced gender ratio; both samples also differed in age with the first sample being older than the second one. These demographic differences must be kept in mind when interpreting the results and further investigations in representative samples must follow. However, presenting at least partly consistent results across the two samples increases the generalizability of our findings thereby offering an evidence-based explanatory framework for the link between empathy and negative affect. The smallest profile in Study 1 is exceedingly small (n = 11), which may cast doubt on the validity of the four-profile solution. Nevertheless, the statistical parameters and theoretical considerations indicate a strong rationale for maintaining the four-profile solution, incorporating the (A−/C−) profile. The modest proportions of the profile could be attributed to two factors: the small total sample size and the pronounced gender imbalance, characterized by an overwhelming preponderance of female participants. The gender ratio could provide a potential explanation, as the (A−/C−) profile has been observed to contain an above-average number of men and a below-average number of women in both samples. In addition, the profile is primarily small in absolute terms, which could be due to the rather small sample in Study 1. In the literature, profiles with a size of 4% are reported more commonly^29^.

A further limitation concerns the measurement of affective empathy. There has been some debate about whether and how affective empathy can be validly measured^49^. Rather than measuring empathy itself, measures of affective empathy may capture the emotional response to social stimuli^50^. This challenges the theoretical model on which this paper is based, as it is not the level of affective empathy but the ability to regulate emotions that may explain the correlation with depression and anxiety. According to the model proposed by Stevens and Taber^50^, in situations that elicit affective empathy, people respond with either personal distress or empathic concern in the case of unsuccessful or successful self-regulation, respectively. Empathic concern, in turn, enables cognitive empathy. This sequential model, however, appears to stand in partial tension with the independence of affective and cognitive empathy as proposed by Smith’s model, since in the sequential model affective empathy is a precursor to cognitive empathy. In the context of the sequential model, a higher correlation of empathic concern with perspective taking than with personal distress would be anticipated. However, this expectation is not supported by the data presented here or by previous studies^32,33^. Furthermore, previous research, particularly ecological momentary assessment studies, has shown that affectivity and subsequent emotion regulation, as well as empathy, may operate concurrently, with each having its own distinct predictive value for negative affect^51^. Despite these considerations, the different assumptions made by the models will have to be tested against each other in experiments that are designed for this purpose. The present analyses are not sufficient to provide evidence for the superiority of one of these explanatory models. To further refine the existing theoretical models, we need to empirically compare different structural models for the three empathy-related components: personal distress, empathic concern, and perspective-taking. Specifically, Weisz and Cikara^52^ proposed three potential models (lateral, interactive, and nested) to elucidate the interrelationships among these components, and advocated for explicit research directly comparing these structural models to identify the most appropriate representation of the empathy construct.

Taken together, our results speak to the existence of latent combinatory profiles of empathy that are differentially associated to negative affectivity. They are in line with previous literature suggesting a link between pronounced affective empathic traits and psychopathology^8,9,43,53^. Our results suggest that high affective involvement in the emotional experience of another goes along with elevated negative affectivity with the potential to abolish the positive relationship between cognitive empathy and affectivity. As human other-oriented behavior underlies the principle of multiple determinism^54^, changes in empathy alone cannot explain or predict psychopathological conditions. However, observed variations in empathic traits may offer a taxonomy that may help to identify important starting points for clinical interventions, which—other than the vast majority of introduced interventions—do not uniformly call for generally increased empathy but target the differential enhancement and reduction or specific aspects of empathy, respectively. Further research is needed, to uncover potential explanatory mechanisms that elucidate under which circumstances affective empathy leads to negative affectivity.

## Methods

### Study 1

#### Sample

The first sample came from an educational intervention study in primary schools^27^, which has been reviewed and approved by the Human Subjects Committee of the Faculty of Economics, Business Administration and Information Technology at the University of Zurich (Date of Approval: 2012/09/06) and complied with the Declaration of Helsinki (latest version). Written informed consent was obtained from all participants. Parents were surveyed in 2015 as part of a separate follow-up survey for the purpose of the study presented here. Questionnaires and an unconditional incentive of 20 € were sent by post to the participants. Of the 327 initially contacted persons, 259 returned sufficient information and were included in these analyses. The final sample consisted of 232 female (89.6%) and 27 male (10.4%) participants. The mean age was 42.3 (SD = 6.2) years, ranging from 26 to 75 years. All the participants were parents (primarily mothers) of children in primary school.

#### Instruments

##### Assessment of empathy

Affective components of empathy (i.e. personal distress and empathic concern) and the cognitive component of empathy (i.e. perspective taking) were measured with subscales of the Interpersonal Reactivity Scale (IRI)^14^. Personal distress (PD) describes negative feelings such as an aversive and upsetting state of self-focused attention, whereas empathic concern (EC) assesses feelings of warmth, compassion, and concern in response to others’ distress. Perspective taking (PT) captures the extent to which someone tries to understand situations, viewpoints, and feelings from a perspective other than their own. Each scale consists of seven items that are rated on a 5-point Likert-scale from 0 (does not describe me at all) to 4 (describes me very well). In the sample of the educational intervention study (N = 259), all scales had acceptable internal consistencies (α_personal distress_ = 0.63; α_empathic concern_ = 0.65, α_perspective taking_ = 0.74) and showed low to moderate intercorrelation (r_PD and EC_ = 0.33, r_PD and PT_ = − 0.12, r_EC and PT_ = 0.32).

##### Assessment of negative affectivity

*Depression.* In the educational intervention study, we assessed symptoms of depression with the German version of the Center for Epidemiologic Studies Depression Scale (CES-D)^55,56^. The CES-D is a 20-item general population, self-report dimensional measure of depressive symptoms and associated degree of emotional, motivational, cognitive, somatic, and interpersonal impairment during the previous week. It is characterized by strong psychometric properties, such as good levels of internal consistency (α_CES-D_ = 0.88 in the present sample) and convergent validity with other construct-related measures (i.e., Beck Depression or Inventory of Depressive Symptoms^56^). The items are rated on a 4-point Likert-scale from 0 (rarely or not at all [less than 1 day]) to 3 (most of the time, all the time [for 5–7 days]). Participants rate the items with respect to the last 7 days.

*Anxiety.* Dispositional anxiety was assessed by means of the trait form of the State-Trait-Anxiety-Inventory (STAI)^57,58^. The 20-item trait subscale assesses how individuals felt “generally”. The items are rated on a 4-point Likert-scale from 0 (rarely) to 3 (mostly). This subscale has good construct validity and test—retest reliability. In the present sample the scale indicated excellent internal consistency with an α_STAI_ = 0.91.

#### Statistical analysis

All analyses were carried out with R version 3.6.3 (2020). For the LPA analyses we used the tidyLPA package^59^.

As a first descriptive step, we ran a latent profile analysis (LPA) in order to reveal the hypothesized combinatory groups of affective empathy (i.e. personal distress and empathic concern) and cognitive empathy (i.e. perspective taking) proposed by Smith (A+/C+, A+/C−, A−/C+, A−/C−)^11^. The best LPA solution was identified using theoretical and statistical guidelines. We consulted the Bayesian information criteria (BIC)^60^. Model fit decisions were based on the BIC since simulation studies indicate it to be the most accurate criterion fit index^61^. Here, a difference of at least 2–6, 6–10, or > 10 BIC units between two models indicates “positive”, “strong”, or “very strong” evidence of a statistically meaningful improvement in model fit^28^. In addition, bootstrapped likelihood ratio test (BLRT) and entropy were evaluated to quantify the model fit^29^. Moreover, the relevance of the identified latent profiles also depends on whether they predict other outcomes^62^. Therefore—as a next step—we externally validated the integrative and predictive value of our framework. Here, we compared means between identified latent profiles with one-way analyses of variance (ANOVA) for depression and anxiety. Post-hoc comparisons were conducted using Bonferroni adjusted alpha levels of 0.05.

Using multiple linear regression analyses and simple slope analyses, we examined whether the expected positive association between affective empathy and negative affect (depression and anxiety) was attenuated at high levels of cognitive empathy. We first tested for interaction effects between cognitive and affective empathy. Conditional effects (i.e., simple slopes) were estimated for significant interactions. This provides a better understanding of how the relationship between affective empathy and negative affect changes at different levels of cognitive empathy (i.e.: − 1 SD, mean, + 1 SD). To avoid overestimation of the predictive value of variables in our model, predictors and moderators were standardized^56,57^.

### Study 2

#### Sample

The second sample came from the Longitudinal Resilience Assessment (LORA) study^30^. The study consists of a convenience sample of initially healthy individuals. The sample was drawn from the general population of the cities of Frankfurt and Mainz, Germany. Adults were surveyed as part of the first baseline assessment (B0) at the study centers. Participants were reimbursed with 60 € for the first assessment. From the initial cohort of 1191 participants at study entry, 938 provided full information and were included in these analyses. The final sample included 591 females (63.0%) and 345 males (36.8%). Mean age was 28.6 years (SD = 7.8), ranging from 18 to 50 years. The study was approved by the respective Ethical Committees in Mainz (registration number: 837.105.16 (10,424)) and Frankfurt (registration number: 244/16) and complied with the Declaration of Helsinki (latest version). Written informed consent was obtained from all participants.

#### Instruments

##### Assessment of empathy

Empathy was assessed with the same instrument as in the previous sample (IRI)^14^. In Study 2 (N = 938), all scales had acceptable internal consistencies (α_personal distress_ = 0.70; α_empathic concern_ = 0.75, α_perspective taking_ = 0.77) and showed low to moderate intercorrelation (r_PD and EC_ = 0.37, r_PD and PT_ = − 0.16, r_EC and PT_ = 0.32).

##### Assessment of negative affectivity

*Depression.* In Study 2 we assessed symptoms of depression with the Patient Health Questionnaire—depression subscale^63^. The scale consists of nine items, representing symptoms typical for clinical depression. The items are rated on a 4-point Likert-scale from 0 (not at all) to 3 (almost every day). Participants rate the items with respect to the last 14 days. This scale allows to assess the severity of depressive symptoms. With an α_PHQ-9_ = 0.78 the scale indicated acceptable internal consistency in Study 2 (N = 938).

*Anxiety.* We assessed symptoms of anxiety with the anxiety and insomnia subscale of the General Health Questionnaire (GHQ)^64,65^. This self-report measure assesses whether changes in psychological well-being have occurred in the past 4 weeks. The scale consists of seven items and each item is rated on a 4-point scale from 0 (not at all) to 3 (much more than usual). Participants rate the items with respect to the last weeks (not further specified). With an α_GHQ_ = 0.83 the scale indicated good internal consistency in the sample of study 2.

#### Statistical analyses

We exactly replicated the analyses conducted for the first sample.

## Data Availability

The data of Study 1 have been collected in the context of a larger educational project and thus represents highly sensitive data. This dataset cannot be made available for data protection reasons. In addition, parental consent for data usage did not include data storage in a public repository but only sharing completely anonymized data for scientific purposes and after the collaborating scientist signed a respective research agreement. The restriction to scientific purposes was also necessary to comply with data protection requirements. Similarly, for Study 2, ethical approval and the respective informed consent did not include data storage in a public repository. For both studies, completely anonymized datasets underlying the results of the presents study are available on request from the corresponding author (MW) and after filling out a research agreement.
